# Isolation and identification of soil bacteria capable of degrading biodegradable mulch films

**DOI:** 10.1007/s10532-025-10223-4

**Published:** 2025-11-20

**Authors:** Harshal J. Kansara, Yvan D. Hernandez-Charpak, André O. Hudson, Thomas A. Trabold, Jeffrey S. Lodge, Carlos A. Diaz

**Affiliations:** 1 Rochester Institute of Technology, Golisano Institute for Sustainability, Rochester, NY USA; 2 Thomas H. Gosnell School of Life Sciences, Rochester Institute of Technology, Rochester, NY USA; 3 Department of Packaging and Graphic Media Science, Rochester Institute of Technology, Rochester, NY USA; 4 Department of Mechanical Engineering, Universidad de Los Andes, Bogotá, Colombia

**Keywords:** Culture enrichment, Biodegradable mulch films (BMFs), Bacterial growth, Carbon mineralization, Biodegradation

## Abstract

Agricultural mulch films (AMFs) enhance crop productivity by controlling soil temperature and moisture and suppressing weed growth. Conventional AMFs made from polyethylene (PE) pose disposal challenges and contribute to long-term plastic pollution. Biodegradable mulch films (BMFs) offer a promising alternative, but their degradation in soil remains slow and inconsistent. This study employed a culture-enrichment approach to isolate soil bacteria (i.e., *Pseudomonas guariconensis* and *Achromobacter denitrificans*) capable of accelerating BMF biodegradation. Bioaugmentation with *P. guariconensis* enhanced CO₂ evolution in soil, with 48% and 36% carbon mineralization for two commercial BMFs (i.e., Bio360 and EcoVio), compared to 17% and 6.2% in non-inoculated soils. These findings demonstrate that targeted bacterial enrichment can accelerate BMF degradation, highlighting the potential for bioaugmentation to mitigate plastic accumulation in agricultural soils.

**Graphical Abstract:**

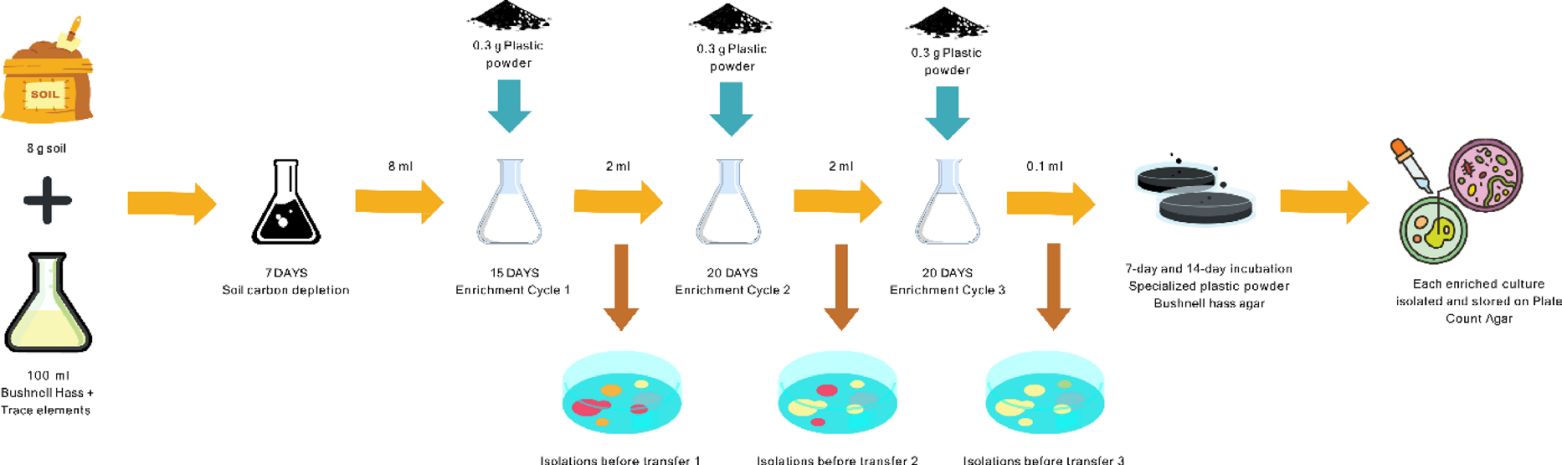

**Supplementary Information:**

The online version contains supplementary material available at 10.1007/s10532-025-10223-4.

## Introduction

Plastic mulch films are widely used in specialized agriculture for their agronomic benefits, including weed suppression, enhanced soil temperature, reduced moisture loss, and improved crop yield and quality (Kasirajan and Ngouajio 2012a; Li et al. 2014). Conventional mulch films use non-biodegradable polymers such as polyethylene (PE) (Abduwaiti et al. 2021). Over the cropping season, they become contaminated with soil and chemicals, complicating recycling (Brodhagen et al. 2015a). As a result, most are landfilled (~ 50% globally), incinerated, or mechanically recycled (Kasirajan and Ngouajio 2012b; Razza et al. 2020). Further, at the end of every harvest cycle, films must be manually pulled from the site to be landfilled. Manual removal post-harvest often leaves plastic fragments behind, contributing to soil contamination and long-term environmental concerns (Madrid et al. 2022). In China, accumulation of PE mulch residues in topsoil has exceeded 240 kg ha⁻^1^, impairing soil health and crop productivity (Gao et al. 2019a). This phenomenon termed “white pollution” has been extensively documented in regions like Xinjiang, where persistent mulch debris continues to affect farmland (Liu et al. 2014; Steinmetz et al. 2016; Gao et al. 2019b).

Biodegradable mulch films (BMFs) are designed to perform comparably to conventional PE films while degrading in situ through natural soil processes such as weathering and microbial action, ultimately mineralizing into CO₂, H₂O, and biomass (Kasirajan and Ngouajio 2012b). Most commercial BMFs are formulated from biodegradable polyesters e.g., poly(lactic acid) (PLA), poly(butylene adipate-co-terephthalate) (PBAT), poly(caprolactone) (PCL), and poly(butylene succinate) (PBS) or polysaccharides like thermoplastic starch (TPS), chitin, and cellulose (Menossi et al. 2021; Akhir and Mustapha 2022). Manufacturers optimize blends of these materials to achieve both soil biodegradability and adequate field performance (Mansoor et al. 2022). With increasing awareness of their environmental advantages, the BMF market is projected to grow at 8.4% annually from 2022 to 2027 (Huang et al. 2023). Despite growing interest, the short- and long-term effects of BMF residues on soil and ecosystems remain poorly understood (Sintim and Flury 2017; Liu et al. 2022). While some films meet general standards for aerobic biodegradability (e.g., ASTM D6400 or ASTM D5988), these do not specifically evaluate BMFs in soil environments (Sintim and Flury 2017). EN 17033 (2018) is the only standard tailored for BMFs, requiring 90% degradation in soil at 20–28 °C within two years (Sintim et al. 2020). However, laboratory conditions rarely reflect field realities. Films marketed as “compostable” or “soil biodegradable” often show only partial mineralization post-tillage due to variable soil conditions (Sintim and Flury 2017; He et al. 2018; Sintim et al. 2020). Moreover, micro- and nano-plastics can persist in soil for extended periods (Colwell et al. 2023). A study assessed BMF degradation in agricultural soils in Tennessee and Washington, reporting 61–83% surface-area loss over 36 months in Tennessee but only 26–63% in Washington (Sintim et al. 2020). The lower degradation in colder climates highlights the variability of BMF breakdown under field conditions. These findings underscore the need to accelerate biodegradation rates, particularly in regions with extended winters and slower microbial activity.

The enrichment culture technique is a proven method for isolating polymer-degrading microorganisms. For instance, *Rhodococcus* strain A34 capable of degrading PE from weathered plastic waste (Tao et al. 2023). Enrichment allows selective growth of bacteria that can use polymers as their sole carbon source, effectively excluding non-degraders (Howard et al. 2023). However, most previous studies focused on single-polymer films. Since BMFs are often formulated with polymer blends and contain additives like dyes and fillers (Yu et al. 2024), their use in enrichment cultures presents unique and underexplored challenges.

BMFs are intended to decompose in soil but often persist for extended periods, leaving residual fragments and microplastics that accumulate over time (Bandopadhyay et al. 2018). Microorganisms capable of degrading synthetic polymers are typically isolated from plastic-contaminated or compost environments rather than from agricultural soils, where such polymers remain less familiar to native microbial communities (Zhou et al. 2023; Ali et al. 2023; Nademo et al. 2023). This study aimed to develop a methodology for isolating, identifying, and evaluating soil-resident bacteria capable of colonizing agricultural soils and degrading BMFs using an enrichment-culture approach. We hypothesized that targeted bioaugmentation with these isolates can enhance BMF mineralization by promoting early polymer cleavage into low-molecular-weight oligomers and monomers that stimulate broader microbial activity, providing a scalable strategy to accelerate in situ degradation and mitigate the long-term accumulation of residual film fragments and microplastic particles in agricultural soils.

## Materials and methods

### Mulch films and polymers

The study tested three agricultural mulch films (AMF):

a) Conventional low-density polyethylene (LDPE) film from Ken-Bar Mulch (Rochester, NY, USA);

b) BMF from Organix A.G. (Bloomington, MN, USA), composed of BASF EcoVio (EV), a PLA and PBAT blend (Mena-Prado et al. 2023), and.

c) A BMF from Dubois Agrinovation (Saint-Rémi, QC, Canada), containing Bio360 (B360), a blend of thermoplastic starch (TPS), poly(caprolactone) (PCL), and PBAT (Ruggero et al. 2020a).

LDPE was used as a negative control, while EcoVio (EV) and Bio360 (B360) served as test films. PBAT (EcoWorld®) was sourced from Jinhui Zhaolong (China), PLA (grade L175) from TotalEnergies Corbion (Netherlands), and PCL Capa 6800 from Perstorp (UK). Starch was obtained from MP Biomedicals (USA), and thermoplastic starch was prepared using a CWB Brabender Intelli-Torque mixer with a 60 cc, 3-piece mixing head.

### Soil inoculum

Commercial “Scotts Bovung Manure Blend” (Dearborn, MI, USA), composed of matured compost and manure, was used as the soil inoculum for bacterial isolation. Detailed soil analysis is available in Table S1 and in Bhattacharya et al. (Bhattacharya et al. 2023a). The abundance and diversity of cultivable microorganisms were assessed using plate count agar (PCA) (Difco™, Becton Dickinson, USA; Lot No. 9349812) for bacteria and potato dextrose agar (PDA) (Difco™, IBI Scientific; Lot No. 11F1850) supplemented with streptomycin sulfate (100 µg/mL) for fungi. Both media showed high colony counts at 30 °C (Figure S1). Morphological assessment revealed at least five distinct fungal colonies on PDA + SS and nine bacterial colonies on PCA after two days. This soil has been previously characterized and validated as a suitable microbial source for plastic degradation studies (Bhattacharya et al. 2023a).

### Isolation and growth media

Enrichment cultures were grown in Bushnell Hass mineral medium (BHM; HiMedia, India; Lot No. 0000117466), a carbon-free selective medium (pH 7.0 ± 0.2). Tryptic soy broth (TSB; EMD Millipore, USA; Lot No. VM987359 132) was used as a rich growth medium (pH 7.3 ± 0.2). Trace elements (TE, 1 µL/mL), based on the formulation by Hayase et al. (Hayase et al. 2016), were added to all BHM media (see Table 1) to support key bacterial metabolic functions, including enzymatic cofactor activity, redox regulation, and detoxification (Begg 2019).

**Table 1:** Chemical composition of the trace elements used in all BHM media and BHM agar plates

Chemical component	Composition (mg/mL)
CoCl_2_.6H_2_O	11.9
NiCl_2_	11.8
CrCl_2_	6.3
CuSO_4_.5H_2_O	15.7
FeCl_3_.6H_2_O	970
CaCl_2_.2H_2_O	780
MnCl_2_.4H_2_0	10

**Fig. 1:**
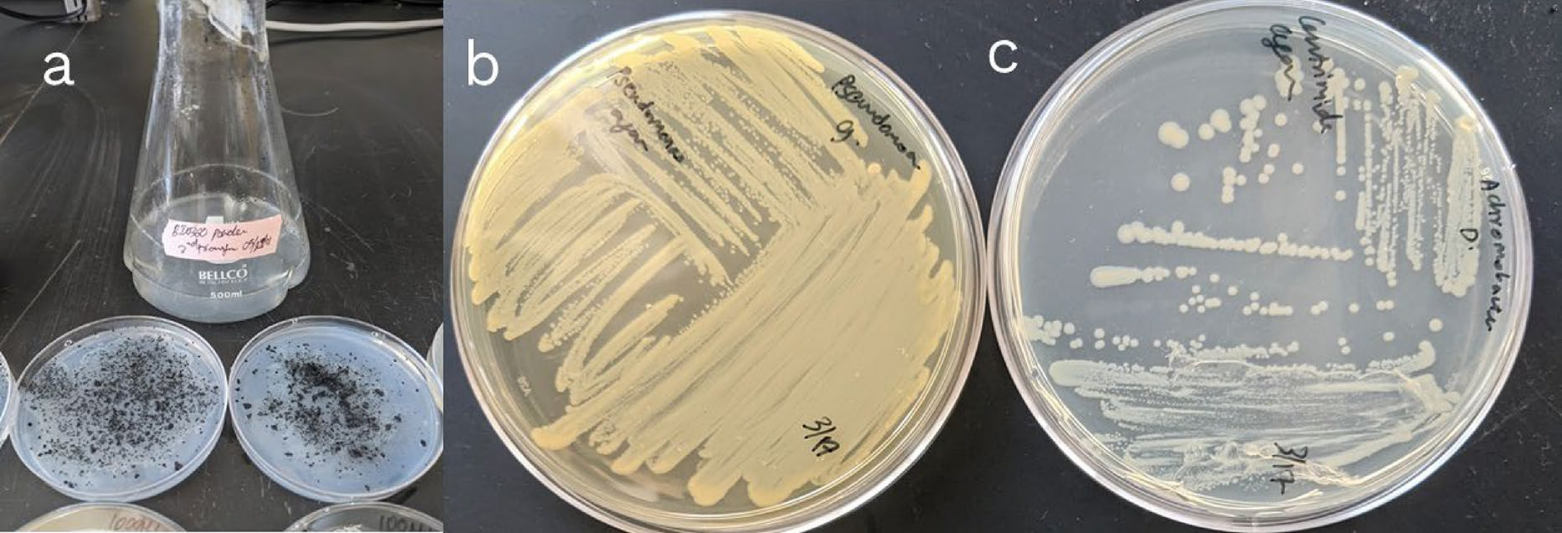
Specialized BHM agar containing BMF powder uniformly on the surface were made. **a** shows B360 mulch powder on BHM + TE agar. The bacterial cultures were then inoculated and incubated on the specialized agar. The bacterial colonies were carefully selected using a sterile needle, isolated and purified as shown in **b** (Pseudomonas sp. R40.2) and **c** (Achromobacter denitrificans strain 411)

After enrichment, specialized BHM + TE agar plates were prepared by autoclaving the media and then evenly coating the surface with BMF polymer powders (Fig. 1), since autoclaving the polymers with agar led to softening and clumping. These plates enabled selective isolation of polymer-degrading bacterial colonies. BHM, TSB, and PCA media were sterilized via liquid autoclave at 121.5 °C for 15 min. Isolates were stored on PCA at 4 °C and maintained by biweekly transfers.

### Microplastic powder preparation

Film cryogrinding was conducted using a SPEX SamplePrep freezer mill 6870 (Metuchen, NJ, USA). Films were cut into 2 × 6 cm strips and milled in three rounds, each using > 5 g of material. Each round involved three 10-min grinding cycles, with 2-min cooling intervals. Prior to milling, films were cleaned with deionized water and 95% ethanol, then air-dried. Ground powders were transferred to sterile plastic pouches, sealed, and stored at room temperature.

### Enrichment of cultures and isolation

Eight grams of soil were added to 100 mL of carbon-free BHM + TE in a 250 mL Erlenmeyer flask and incubated at 30 °C, 150 rpm for 7 days to deplete readily available native carbon, thereby promoting selective enrichment of microorganisms capable of utilizing the introduced polymer as the principal carbon source. Then, 8 mL of this inoculum was transferred to 100 mL of fresh BHM + TE in a 500 mL flask, along with 0.3 g of sterile plastic powder. The flask was incubated for 15 days under the same conditions. This enrichment was repeated through two additional transfers, each using 2 mL of inoculum, 0.3 g of plastic powder, and 100 mL of fresh BHM + TE, with 20-day incubation periods at 30 °C, 150 rpm. The process was performed for all polymer types.

After enrichment, 100 µL of the final culture was spread onto BHM + TE agar coated with polymer powder (Figure S1) and incubated up to 14 days, with colony picks at days 7 and 14 to capture both fast- and slow-growing isolates. Resulting colonies were purified and transferred to PCA plates (30 °C, 24 h) and maintained by biweekly transfers. The enrichment procedure is summarized in Figure S2.

### 16 s rRNA sequencing

Enriched strains were identified by polymerase chain reaction (PCR) mplification of the V3 region of the 16S rRNA gene. Genomic DNA was extracted by transferring a small amount of each isolate from PCA plates into 1.5 mL microfuge tubes containing 100 µL nuclease-free H₂O. Tubes were heated at 95 °C for 5 min using a SimpliAmp thermal cycler (Thermo-Fisher Scientific, USA), then cooled and stored at 4 °C.

PCR reactions included 1 µL each of V3-forward (5′-ACTCCTACGGGAGGCAGCAG-3′) and V3-reverse (5′-ATTACCGCGGCTGCTGG-3′) primers, 12.5 µL Taq master mix, 10.5 µL nuclease-free H₂O, and 2 µL of DNA extract. Cycling conditions were: initial denaturation at 95 °C (1 min), followed by 30 cycles of 94 °C (45 s), 50 °C (30 s), and 72 °C (30 s), and final extension at 72 °C for 5 min. Products (~ 200 bp) were stored at 4 °C, cleaned using the EZ-10 PCR cleanup kit, and quantified with a NanoDrop One spectrophotometer. Sanger sequencing was performed by GENEWIZ (Azenta Life Sciences, USA), and taxonomic identification was conducted using the National Center for Biotechnology Information (NCBI) database via the Basic Local Alignment Search Tool (BLAST).

### Bacterial growth curves

A loopful of each isolated bacterial culture from a stock plate of PCA was transferred into 25 mL of TSB in 125 mL Erlenmeyer flasks and incubated at 30 °C, 150 rpm for 24 h. One additional flask was prepared containing a mixture of isolates from each BMF. After incubation, 10 mL of culture was centrifuged (Allegra 6R, Beckman Coulter, USA) at 3750 rpm for 10 min. The supernatant was discarded, and the cell pellet was washed twice with Bushnell Hass mineral medium (BHM) to remove residual nutrients, then resuspended in 10 mL of BHM. Next, 0.5% (w/v) powdered plastic was added to 100 mL BHM with trace elements (TE) in a 500 mL flask. A 1% (v/v) inoculum of cleaned cells was introduced. Every five days, 100 µL was sampled, serially diluted, and plated onto PCA. Colony counts were recorded after 48 h at 30 °C and expressed as $${log}_{10}[(colony forming units \left(CFU\right)]/ml$$. Baseline controls without carbon source were run in parallel.

### Bioaugmented soils setups

Specific Bioaugmented soils were soils were developed for the carbon mineralization testing and the procedure is as follows: A loopful of *P. guariconensis* was inoculated into 10 mL of TSB in a 125 mL flask and incubated at 30 °C for 24 h. The culture was scaled up to 250 mL in a 1 L flask and incubated for an additional 48 h. After incubation, cultures were diluted and plated to CFU counts and absence of contamination. Fresh “Scotts Bovung Manure Blend,” the original isolation source, was sieved to < 0.5 cm particle size. A high-density inoculum (10⁹ CFU/kg soil) from the flask was sprayed onto the soil and mixed thoroughly. Moisture was adjusted to 50–60%, and this was designated as enhanced soil (ES). A parallel batch, prepared identically but without inoculum, was designated as baseline soil (BS). A similar methodology was used for all bacterial cultures.

### Carbon mineralization tests

Biodegradation was quantified in sealed biometer flasks using the titrimetric CO₂ evolution method, following established procedure (Andrady and Song 1999; Chiellini and Corti 2003; Dangi et al. 2021), using the ES and BS. CO₂ evolution refers to the biological release of carbon dioxide resulting from microbial mineralization of biodegradable polymers in closed biometer systems. A schematic of the setup and calculation workflow is provided in Figure S3 and detailed in Section S3 of the Supplementary Information.

### Microcosm jar experiments

Fine perlite (100 g) was evenly distributed in a clean 1 L jar as a base layer and pre-moistened with 20 mL of deionized (DI) water. A 50 g layer of sieved soil was added, followed by careful placement of a 7.62 cm^2^ BMF sample. An additional 50 g of ES or BS was layered to bury the film completely. The surface was topped with 10 g of coarse perlite, lightly moistened with 10 mL DI water to retain surface humidity. Jars were covered with breathable cheesecloth to permit gas exchange and prevent contamination. Each setup was weighed to 1 g precision for moisture tracking. Jars were incubated at 30 °C, with periodic weighing and water replenishment to maintain soil moisture. See Figure S4 for setup illustration.

Each jar represented a time point for bacterial quantification at 0, 60, 120, and 180 days. As films degraded, BMF samples were recovered at 60 and 120 days, rinsed with DI water, and dried overnight at 60 °C. Additionally, 1 g of surrounding soil was collected before film cleaning and diluted to determine bacterial counts using PCA.

## Results and discussion

In the following sections, results are presented alongside interpretive analysis to provide context and align with study hypotheses. This integrated approach avoids redundancy and ensures each finding is directly connected to its scientific implications.

Preliminary soil-burial experiments were conducted to assess whether native soil microbial communities could colonize and degrade pristine BMFs. However, due to the hydrophobic nature of pristine BMFs, negligible mass loss was observed over 200 days. Moreover, assessments of bacterial and fungal diversity using PCA and PDA with streptomycin sulfate (SS), respectively, showed no noticeable changes. Results of these preliminary trials are shown in Figure S5.

“BMF weathering” refers to the environmental degradation of films in the field due to factors such as solar radiation, temperature fluctuations, and soil moisture (Anunciado et al. 2021). These conditions gradually weaken the polymer matrix, making it more susceptible to embrittlement and subsequent biodegradation (Hablot et al. 2014; Anunciado et al. 2021). A preliminary study on BMF weight loss due to weathering and its impact on microbial isolation is presented in Figure S6.

Studies have shown that BMF-degrading microbes secrete extracellular enzymes that cleave polymer chains into monomers and oligomers, which can then be metabolized by other soil microorganisms (Brodhagen et al. 2015b; Bandopadhyay et al. 2020). Although burial studies allowed observation of community shifts, they did not enable specific isolation of cultures responsible for initiating BMF hydrolysis. In passive soil burial, abiotic hydrolysis and community cross-feeding can support growth of many non-degraders on released monomers/oligomers, making it difficult to attribute polymer cleavage to specific taxa (Montazer et al. 2020; Wicaksono et al. 2022). We therefore adopted an enrichment-culture strategy, with polymer as the sole added carbon source and repeated transfers, followed by isolation on polymer-coated plates to recover putative primary degraders.

### Enrichment of cultures and 16S sequencing results

Culture enrichment was conducted using selected polymer powders as the sole carbon source in BHM + TE. All enrichment cultures in this study were derived from a single commercial soil blend to maintain consistency across experiments. While this allowed controlled comparisons, it may limit generalizability to other soil types; this limitation is addressed further in ‘Limitation and Future work’ section. An initial 7-day soil carbon depletion step preceded enrichment. Post-depletion plating on PCA revealed six distinct bacterial colonies, each with a density exceeding 1 × 10⁶ CFU.

Following the first 15-day enrichment cycle, colony diversity decreased. The LDPE flask yielded three morphologically distinct colonies on PCA, while the B360 and EV flasks each yielded four. Two additional enrichment cycles (20 days each) further refined the communities. Final plating revealed two dominant morphotypes per film type.

Specialized BHM + TE agar plates coated with plastic powders were then used to selectively isolate polymer-degrading colonies (Fig. 1a). Each BMF type consistently yielded two dominant colony types. Examples of isolates recovered from B360 enrichment are shown in Figs. 1b and 1c. Final isolates were subjected to 16S rRNA gene sequencing. Taxonomic identification results are presented in Table 2.

**Table 2:** Identification of isolates from the Ken-Bar mulch (LDPE), Dubois Agrinovation (Bio360), Organix A.G. (EcoVio)

Culture genus and spp.	Film material tested
*Achromobacter denitrificans*	Ken-Bar (LDPE)
*Pseudomonas guariconensis*	
*Achromobacter denitrificans strain 411*	Dubois Agrinovation (Bio360)
*Pseudomonas sp. R40.2*	
*Achromobacter denitrificans*	Organix A.G. (EcoVio)
*Pseudomonas guariconensis*	

### Effect of increased carbon availability on culture growth

Biodegradable polymer microplastics can alter soil chemistry, microbial communities, and crop productivity, as discussed by (Malafeev et al. 2023). This experiment aimed to evaluate how increasing carbon availability via different concentrations of Organix A.G. (EV) BMF powder affects the growth of enriched bacterial isolates in BHM + TE.

EV powder was selected over B360 due to its greater hydrophobicity, which was hypothesized to impact polymer accessibility and microbial response. Cryo-ground EV powder was tested at 0.5%, 1.0%, and 2.5% (w/v). Enrichment isolates from the EV culture were first grown in TSB for 24 h, harvested by centrifugation, and washed three times in BHM. A 0.5% (v/v) inoculum was added to 500 mL flasks containing 100 mL of BHM + TE and the respective polymer concentrations. Growth was assessed every five days by plating serial dilutions from 100 µL aliquots onto PCA. The experimental workflow is illustrated in Figure S7.

**Fig. 2:**
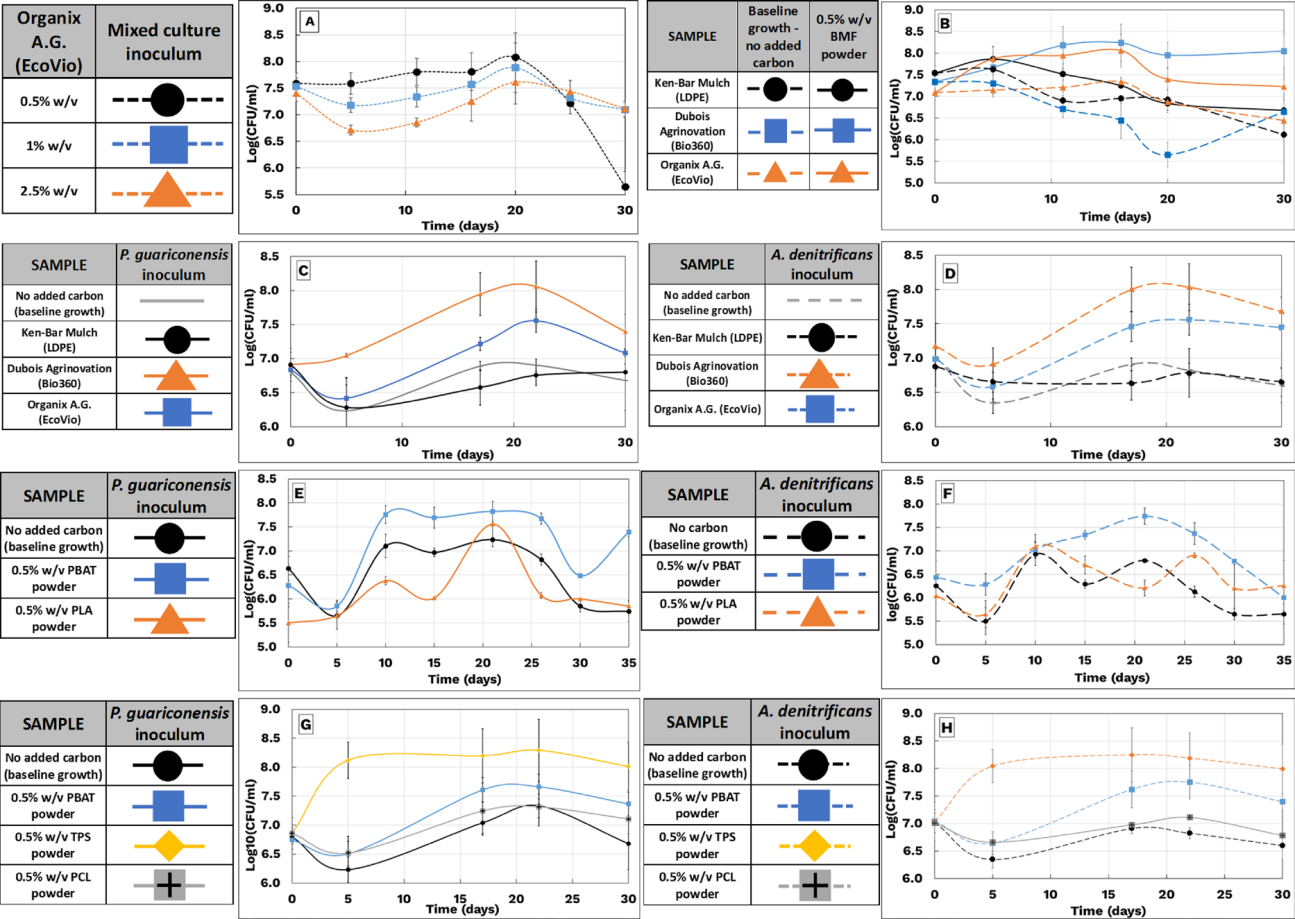
Time-series bacterial growth on bio-based mulch films (BMFs) and constituent polymers. **A** Effect of increasing carbon loading on mixed cultures (A. denitrificans + P. guariconensis; 0.5% v/v each). **B** Growth of mixed cultures on BMFs: EV (Organix A.G.), B360 (Dubois Solution), and LDPE (KenBar). **C** P. guariconensis growth on BMF powders from EV, B360, and LDPE. **D** A. denitrificans growth under the same BMF powder conditions. **E** P. guariconensis growth on PBAT and PCL, inoculated from EV enrichments. **F** A. denitrificans growth on PBAT and PCL, inoculated from EV enrichments. **G** Pseudomonas spp. R40.2 growth on PBAT, PCL, and TPS, derived from B360. **H** A. denitrificans strain 411 growth on the same polymer substrates as in (**G)**. In all panels, cultures were grown in BHM + TE medium supplemented with 0.5% w/v BMF or polymer powders. Data represent mean colony counts over time, with error bars denoting ± 1 standard deviation from triplicate dilutions plated on PCA

As shown in Fig. 2A, the flask containing 0.5% EV powder exhibited a 0.6-log₁₀ increase in colony counts by day 20, followed by a sharp decline, suggesting possible nutrient limitation or oxygen restriction, although nutrient concentrations were not directly measured. In contrast, flasks with 1.0% and 2.5% EV powder showed initial reductions in CFU by 0.3 and 0.7 logs, respectively, before recovering slightly, reaching net increases of 0.3 and 0.1 logs.

The counterintuitive reduction in growth at higher carbon concentrations is attributed to the poor dispersion of the hydrophobic polymer. At 1.0% and 2.5%, the powder formed a mat on the media surface, likely reducing carbon accessibility and oxygen transfer. Over time, partial submersion and microbial adaptation may have enabled limited recovery.

**Fig. 3:**
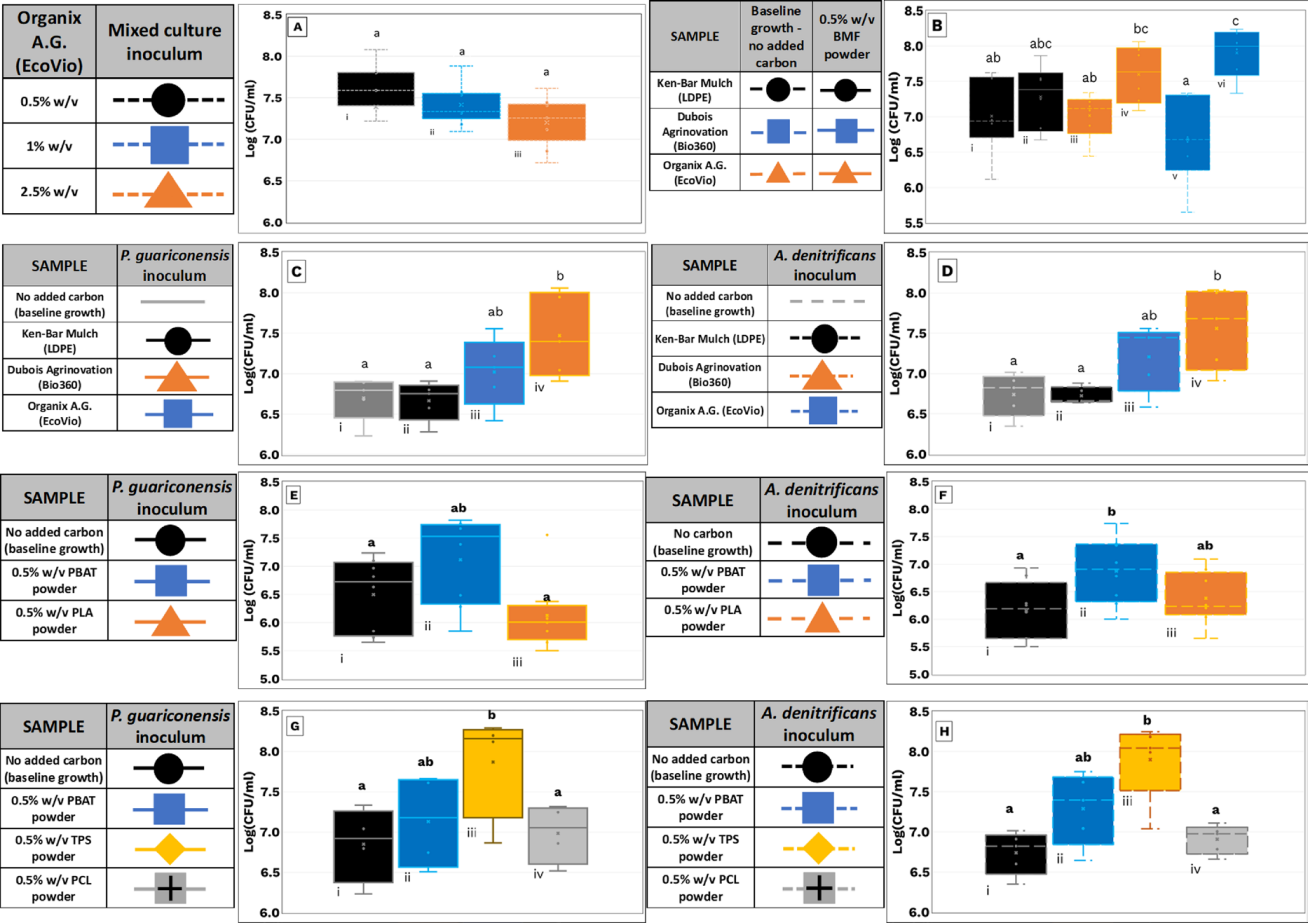
Statistical distribution of bacterial growth under BMF and polymer substrate conditions. Boxplots display the mean bacterial growth and variability for corresponding conditions in Fig. 2. The lower and upper hinges of each box represent the 25th and 75th percentiles; whiskers extend to 1.5 times the interquartile range. Letters above the boxes indicate Tukey post hoc groupings following one-way ANOVA (*α* = 0.05). Data are derived from three independent colony count dilutions. **A** Carbon loading impact on mixed culture growth (A. denitrificans + P. guariconensis). **B** Mixed culture bacterial growth on EV, B360, and LDPE BMFs. **C** P. guariconensis growth on BMF powders from EV, B360, and LDPE. **D** A. denitrificans growth under the same BMF powder conditions. **E** P. guariconensis growth on PBAT and PCL. **F** A. denitrificans growth on PBAT and PCL. **G** Pseudomonas spp. R40.2 growth on PBAT, PCL, and TPS. **H** A. denitrificans strain 411 growth on PBAT, PCL, and TPS

Although no statistically significant differences in mean CFU were observed among the treatments (Fig. 3B), previous studies have shown that high carbon or microplastic loadings can suppress microbial activity as noted by (Li et al. 2023). Hence, a concentration of 0.5% (w/v) was selected for subsequent assays to provide adequate substrate without potential inhibitory effects.

### Mixed culture growth curve

To assess the ability of enriched bacterial isolates to grow on BMFs as a sole carbon source, a growth curve experiment was conducted. Two isolates each from LDPE (Ken-Bar), B360 (Dubois Agrinovation), and EV (Organix A.G.) enrichments were first grown in TSB for 24 h. Cells were harvested, washed with BHM + TE, and reinoculated into 500 mL flasks containing 100 mL of BHM + TE with respective BMF powders. Control flasks (no carbon source) were run in parallel. CFU were measured every five days over 30 days via plating on PCA.

As shown in Fig. 2B, EV flasks exhibited a 0.97-log₁₀ increase in CFU by day 16, while B360 flasks increased by 0.85 logs. Both declined afterward, likely due to reduced nutrient or oxygen availability, although this was not directly quantified. LDPE flasks showed a continuous decline, matching the trend in their control, suggesting no carbon utilization. The Tukey test revealed that (A) No significant difference between LDPE and control (Fig. 2B: i vs ii), (B) No difference between EV and control (iii vs iv), and (C) A highly significant difference (p < 0.01) between B360 and its control (v vs vi).

B360 contains TPS, PCL, and PBAT (Ruggero et al. 2020b). TPS, being hydrophilic (Soares et al. 2013), may reduce film hydrophobicity and promote faster dispersion and microbial access. Prior studies show TPS enhances biodegradability compared to polyesters like PBAT and PCL (Bandopadhyay et al. 2020).

EV films (PLA + PBAT) degrade slowly in soil due to low ambient temperatures and PLA’s high glass transition temperature (Ghimire et al. 2020a; Brdlík et al. 2021). Furthermore, EV-based mulches are known for their hydrophobic characteristics. Previous work by Bhattacharya et. al. involved using gliding arc plasma to enhance the surface adhesion of the Organix A. G. mulch (EV). Its hydrophobic nature also impairs carbon availability. A study by (Bhattacharya et al. 2023b), showed improved degradation of EV film after plasma surface treatment, using *P. guariconensis* an isolate from this study.

A Fisher LSD test also found a significant difference between EV and control flasks (Figure S8). However, as Fisher’s method does not correct for Type I errors, the more conservative Tukey test remains preferable (Pereira et al. 2015).

In contrast, no statistical difference was observed between LDPE flasks and controls, confirming that these isolates could not utilize PE as a carbon source.

### Growth of individual isolated bacterial cultures on bmfs

The ability of *P. guariconensis* and *A. denitrificans* to utilize BMFs as carbon sources was evaluated via growth curve experiments. Isolates obtained from enrichments on LDPE (Ken-Bar), B360 (Dubois Agrinovation), and EV (Organix A.G.) were first cultured in TSB for 24 h, then washed and reinoculated into BHM + TE containing the respective BMF powders. Control flasks without a carbon source were prepared for each condition. Bacterial growth was monitored over 30 days via CFU counts on PCA, sampled on days 5, 16, 22, and 30.

As shown in Fig. 2C, P*. guariconensis* exhibited a 1.2-log increase in B360 flasks and 0.8-log increase in EV flasks by day 22, followed by a decline likely due to nutrient exhaustion. LDPE flasks showed only a minor increase, similar to control levels, indicating minimal utilization of LDPE as a carbon source. Box-and-whisker plots summarizing the 30-day experiment are shown in Fig. 3C. One-way ANOVA revealed significant growth on B360 compared to LDPE, but no statistically significant difference between B360 and EV, or between EV and LDPE.

Similarly, *A. denitrificans* showed 0.8-log and 0.6-log increases in B360 and EV flasks, respectively (Fig. 2D), with no growth enhancement in LDPE flasks relative to controls. These findings are summarized in Fig. 3D. Statistical analysis again showed significantly higher growth on B360 than on LDPE, but no significant differences between B360 and EV or EV and LDPE.

Together, these results confirm that both isolates can utilize B360 and EV as carbon sources, while LDPE remains recalcitrant. The presence of thermoplastic starch (TPS) in B360 may enhance initial hydrophilicity and promote faster microbial access, as noted previously. Meanwhile, the slower degradation of EV may be due to its high PLA content and greater hydrophobicity.

### Growth on PBAT and PLA (individual constituents of EcoVio)

Polyesters such as poly(lactic acid) (PLA) and poly(butylene adipate-co-terephthalate) (PBAT), often blended with starch, are widely used in BMFs (Serrano-Ruíz et al. 2018; Bandopadhyay et al. 2020). These blends enhance mechanical properties such as modulus and toughness, which are essential for field performance (Su et al. 2020). However, the influence of such blends on biodegradation remains uncertain. For example, pure PLA has shown no degradation in soil after 12 months, whereas PBAT-PLA blends have degraded by ~ 50% within 200 days under the same conditions (Nomadolo et al. 2022). Understanding the growth of enriched cultures on individual polymer components offers insights into microbial carbon preferences and potential dominant degraders.

To investigate this *P. guariconensis* and *A. denitrificans* isolated from EV mulcwere tested for growth on powdered PBAT and PLA in BHM + TE media. Isolates were pre-cultured in tryptic TSB, washed, and then inoculated into separate flasks containing PBAT or PLA. Control flasks lacked any added carbon. Samples were taken every five days for 35 days, and colony counts were determined on PCA.

As shown in Fig. 2E, P*. guariconensis* demonstrated a clear preference for PBAT, with a 1.3-log increase by day 21. Although the PLA flask showed a 2-log increase, its overall growth trajectory was lower than that of the control flask. No significant differences in mean growth were observed among the three conditions (Fig. 3E), but PBAT flask counts consistently exceeded controls throughout the trial. Similarly, *A. denitrificans* grew more robustly on PBAT than PLA (Fig. 2F). PBAT flasks exhibited a 1.3-log increase by day 21, while PLA flasks peaked at a 1.1-log increase by day 10 before plateauing. A significant difference (*p* < 0.05) was observed between PBAT and control flasks (Fig. 3F), suggesting PBAT as a viable carbon source. In contrast, PLA-supported growth was not statistically different from the control.

These results indicate both isolates preferentially utilize PBAT over PLA, aligning with prior reports on the limited biodegradability of PLA under typical soil conditions (Ghimire et al. 2020b; Brdlík et al. 2021).

### Culture growth on PBAT, TPS, and PCL (individual constituents of Bio360)

To assess microbial growth preferences for individual constituents of the B360 film, *P. guariconensis R40.2* and *A. denitrificans strain 411* both isolated from B360 enrichment were tested for growth on powdered PBAT, TPS, and PCL. Cultures were pre-grown in TSB, centrifuged, washed, and inoculated into BHM + TE containing 0.5% (w/v) of each polymer. Control flasks without polymer served as baselines (Aldas et al. 2021; Dubois Agrinovation 2025). *P. guariconensis* exhibited its highest growth on TPS, reaching 8.3 log₁₀ CFU/mL by day 21 (Fig. 2G), followed by PBAT (7.7 log₁₀). Growth on PCL resembled the baseline, with no statistically significant increase. Only the TPS condition showed significant growth relative to the control (Fig. 3G). Although PBAT did not reach statistical significance, elevated cell counts suggest partial utilization.

*A. denitrificans* showed similar behavior, with statistically significant growth on TPS (Figs. 2H, 3H) and higher, but not statistically significant, counts on PBAT compared to control. No notable growth was observed on PCL.

Together, these findings indicate that both isolates can utilize TPS effectively, with weaker responses to PBAT and no observable growth on PCL. The hydrophilic nature and amorphous structure of TPS may contribute to its enhanced accessibility, supporting its role in accelerating early biodegradation in blended BMFs like B360. While these results focus on monoculture isolates, it is important to note that in natural soil systems, plastic degradation often involves cooperative microbial consortia, with different species contributing complementary enzymatic functions (Salinas et al. 2023a).

### Biodegradation studies

Biodegradation was assessed using specialized 250 mL biometer flasks to quantify carbon mineralization via CO₂ evolution, following established protocols (Andrady and Song 1999; Chiellini and Corti 2003; Dangi et al. 2021). Because these closed systems prevent intermediate sampling or film recovery, a parallel microcosm study was conducted using septate Schott jars adapted from previous designs (Han et al. 2021a). Initiated concurrently, this setup incorporated the same BS and ES used in biometer flasks. While the biometer flasks enabled quantitative assessment of CO₂ production, the jar experiments provided qualitative visual evidence of film degradation and allowed soil sampling to monitor microbial population changes over time. Each jar experiment was conducted in a single replicate.

Biodegradation of BMFs was assessed under two soil conditions: BS, consisting of unamended Bovung manure blend, and ES, inoculated with *P. guariconensis*. B360 and EV films were incubated in biometer flasks containing either BS or ES, with carbon mineralization estimated via CO₂ evolution. Control flasks with only soil (no plastic) were included to account for endogenous microbial respiration.

**Fig. 4:**
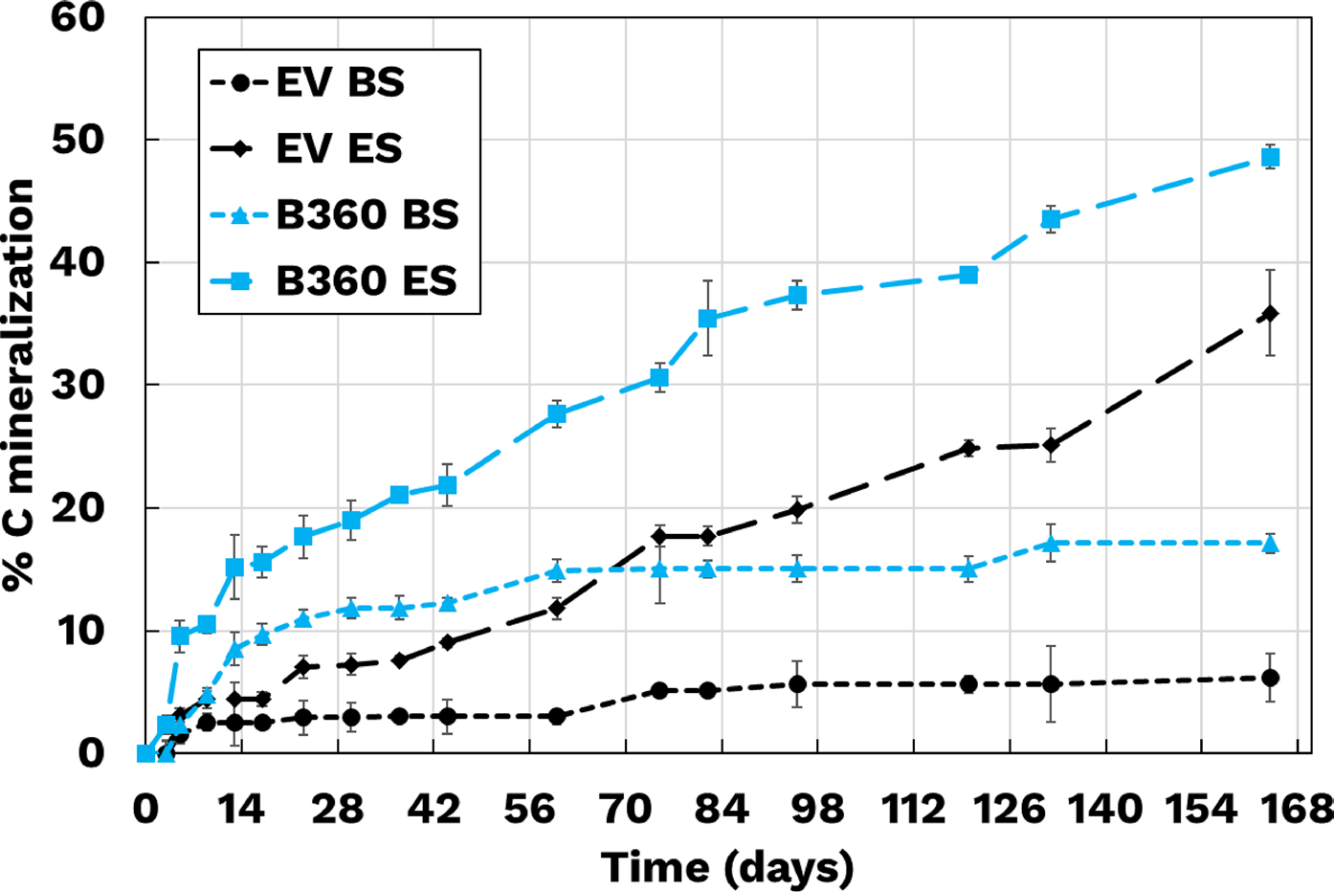
Biodegradation over time (in days) for B360 and EV based BMFs both Enhanced Soil (ES) and Baseline Soil (BS). The error bars represent standard deviation between triplicates. The data indicates that ES inoculated with *P. guariconensis* significantly accelerates the biodegradation of both B360 and EV films compared to baseline soil

As shown in Fig. 4, ES led to significantly greater carbon mineralization compared to BS. For B360, 48 ± 1.1% mineralization was observed after 168 days in ES, versus 17 ± 0.8% in BS. Similarly, EV films reached 36 ± 3.5% mineralization in ES, compared to just 6.2 ± 2.0% in BS. These results demonstrate that enrichment with *P. guariconensis* substantially accelerates film degradation.

These findings are consistent with the hypothesis that bioaugmentation accelerates mineralization by transiently stimulating microbial activity through early-stage polymer cleavage products. In control ES flasks (without BMFs), the enriched bacteria may experience decline due to limited available carbon. In contrast, in ES with BMFs, the release of smaller degradation products supports a synergistic microbial response, leading to increased degradation.

Due to the closed nature of the biometer system, direct validation of this mechanism is limited. To address this, a parallel microcosm study was conducted, allowing for real-time observation and soil sampling during the biodegradation process.

### Microcosm study

The microcosm study simulated controlled soil conditions to assess bacterial activity and film fragmentation during BMF degradation in BS and ES. Identical soil compositions were used across both microcosm and closed-system CO₂ trials, enabling the microcosm to serve as a complementary proxy for observing microbial trends and physical degradation.

**Fig. 5:**
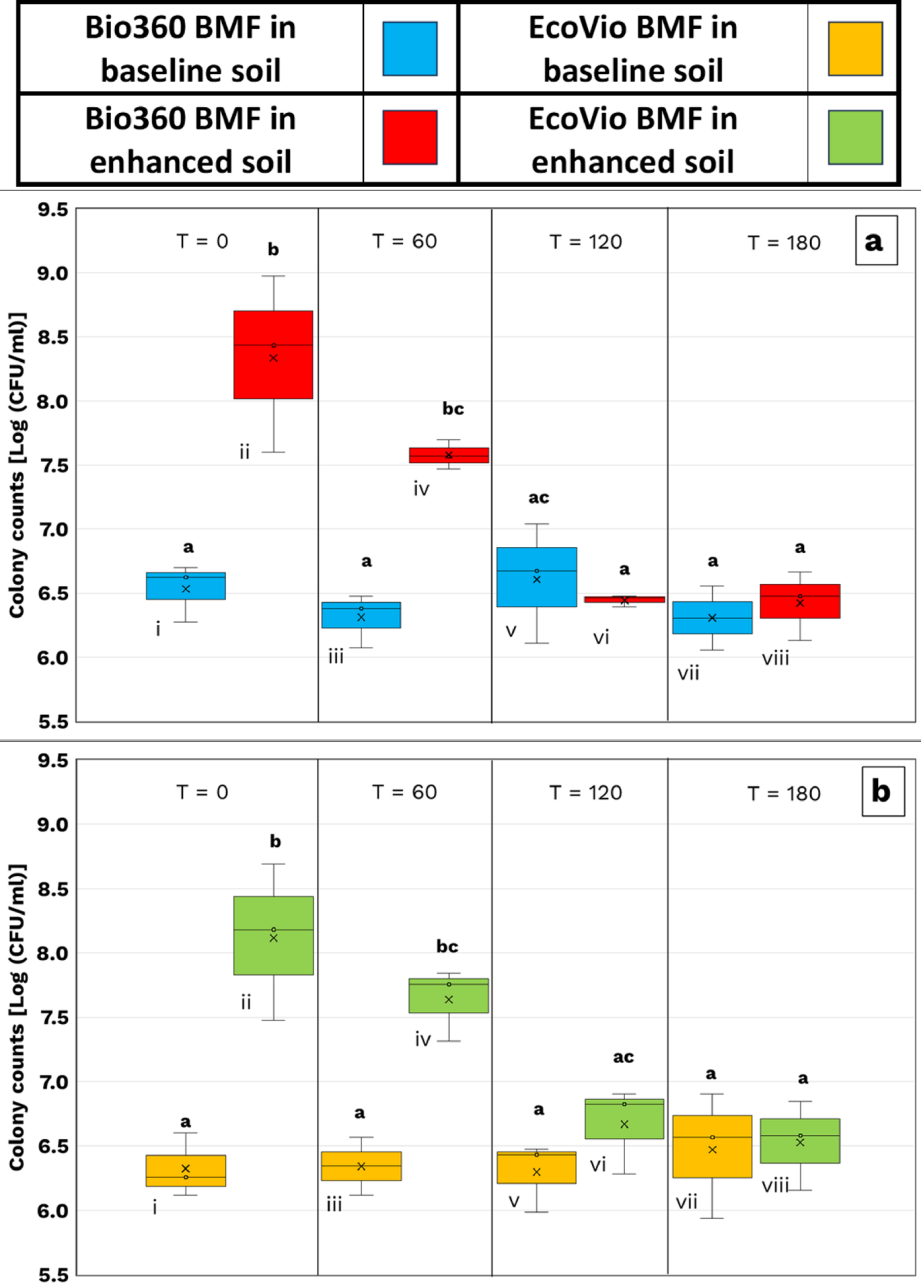
The change in bacterial colony counts (log CFU/mL) over time in baseline soil (BS) and enhanced soil (ES) during the microcosm experiment. **a** presents the bacterial counts for soil samples degrading B360 BMF, while **b** shows the bacterial counts for samples degrading EV BMF. Both Figures represent mean of the bacterial growth cycle in BS and ES soils along with the lower and upper hinges of the boxes representing 25th and 75th percentile, whiskers show 1.5 times inter-quartile range and letters displayed represent Tukey post hoc results following one-way ANOVA with significance tested at *α* = 0.05. The error bars represent 1 standard deviation from mean, derived from three separate dilutions of colony counts isolated on PCA

While the biometer flasks captured CO₂ evolution, the microcosms allowed direct sampling and visual tracking of microbial dynamics. Figure 5a shows bacterial counts in soils with B360 film. Initial ES counts were significantly higher (8.3 ± 0.6 log CFU/g) compared to BS (6.5 ± 0.2 log). By day 60, ES counts dropped to 7.6 ± 0.1, while BS remained stable. By day 120, counts in both soils converged (~ 6.3 log), and this trend persisted through day 180.

A similar trend was observed with EV films (Fig. 5b). ES began at 8.1 ± 0.5 log CFU/g, declining to 7.6 ± 0.2 by day 60, while BS stayed near 6.3 log. Again, counts equalized by day 120 and remained stable through 180 days.

This convergence suggests that the initial microbial stimulation in ES was transient, likely reflecting short-term utilization of oligomeric degradation products released during early stages of film breakdown. Additionally, such high microbial abundances are not typically sustained in soil environments; the initial spike reflects the nutrient-rich TSB inoculum and optimal incubation conditions, which could not be maintained in the soil microcosm. As readily available carbon and nutrients were exhausted, microbial populations in ES stabilized at levels comparable to BS.

ES showed significantly greater and faster biodegradation of B360 and EV films compared to BS. Film weight loss (Figure S9) and visual fragmentation confirmed higher degradation in ES, consistent with greater microbial activity during the early phase of incubation. Although ES initially maintained higher microbial counts, this advantage diminished over time as labile carbon from film degradation was consumed, and cell abundances in both soils converged after day 120.

## Limitations and future work

The enrichment culture technique using BHM + TE has been widely applied to isolate polymer-degrading microbes (Eriksson et al. 2002; Kolsal et al. 2017; Kayath et al. 2019). In this study, BHM + TE was used not only for selective enrichment but also to evaluate the isolates' capacity to utilize BMF polymers as a sole carbon source. While effective for selection, BHM’s minimal composition can result in subtle growth trends that may not yield statistically significant differences relative to controls. Araujo et al. similarly noted reduced diversity in cultures enriched on oil using BHM compared to richer media like yeast extract peptone dextrose (YPD) (Araújo et al. 2020). Although a 1-log shift may reflect substantial microbial changes (∼90% gain or loss), the simplicity of BHM + TE may mask important microbial interactions. Future work could integrate richer media alongside BHM to capture a broader spectrum of polymer-degrading taxa.

The use of a single commercial soil blend limited microbial diversity in this study. Previous research has shown that soil origin strongly influences BMF degradation. For example, a study demonstrated variable PBAT film degradation rates across different soils, linked to distinct microbial communities shaped by physicochemical properties such as pH, texture, organic-matter content, moisture, and nutrient availability (Han et al. 2021b). These factors influence microbial diversity and enzyme expression, ultimately determining BMF degradation efficiency. Similarly, Bandyopadhyay et al. reported significant taxonomic differences in microbial consortia from field soils tilled with BMFs (Bandopadhyay et al. 2020). Their study found significant taxonomic differences in the microbial communities isolated from the two soils (Bandopadhyay et al. 2020). Expanding the range of soils used for enrichment could uncover more efficient or synergistic bacterial strains relevant to diverse agricultural conditions (Salinas et al. 2023b).

Although conducted under controlled conditions, the findings must be viewed in light of real-world variables such as fluctuating temperature, UV exposure, and moisture. These factors can influence both polymer breakdown and microbial performance. For instance, the high glass transition temperature of PLA slows its degradation in cool soils (Karamanlioglu and Robson 2013), while UV and heat exposure can weaken polymer chains, enhancing microbial accessibility (Lindblad et al. 2002). Therefore, future research should test enriched strains under field or simulated field conditions to evaluate their efficacy in real agricultural settings.

Building on this concept, future work could design synthetic microbial communities (SynComs) that integrate complementary degradative pathways from multiple strains. Such consortia may enhance BMF degradation through synergistic enzyme activity, increased substrate range, and greater resilience under variable soil conditions.

Both the biometer flask and microcosm jar approaches offered complementary insights into BMF biodegradation. While biometer flasks enabled quantitative CO₂ monitoring, their closed design limited intermediate sampling. Microcosms, though more labor-intensive, allowed soil sampling and visual observation of film degradation, making them valuable for future strain tracking under semi-field conditions.

Accelerated BMF degradation is vital for reducing plastic persistence in soil, which can degrade soil structure, moisture retention, and crop productivity (De Souza Machado et al. 2019). Microplastic residues from incomplete BMF breakdown may pose long-term ecological risks. This study contributes to sustainable agriculture by identifying bacteria capable of enhancing BMF mineralization. However, further studies are needed to evaluate their effectiveness in variable, real-world field conditions (Cai et al. 2023).

In a future study to further assess the novelty and ecological significance of the isolated bacteria, we plan to perform whole-genome sequencing and comparative genomic analyses. This approach will enable us to identify specific genes and metabolic pathways associated with polymer degradation, including those encoding hydrolytic enzymes such as esterases, cutinases, and lipases. By integrating genomic and proteomic data, we aim to uncover potential mechanisms and modes of action that facilitate the breakdown of complex biodegradable polymer blends like PBAT, PLA, and TPS. These insights could not only elucidate the molecular basis of polymer biodegradation but also reveal new biocatalytic capabilities with potential applications in bioremediation and sustainable materials management. Furthermore, the availability of complete genome sequences will provide a foundation for future transcriptomic and metabolomic studies, helping to clarify how environmental factors influence gene expression during plastic degradation.

## Conclusion

In this study, two distinct bacterial isolates were identified from each of two BMFs and shown to grow using BMF powders as their sole carbon source in BHM. Cultures isolated from EV mulch included *P. guariconensis* and *A. denitrificans*, while those from B360 mulch included *Pseudomonas sp. R40.2* and *A. denitrificans strain 411*. All demonstrated polymer utilization, with significantly greater growth observed on B360 due to its thermoplastic starch (TPS) content. No growth was observed on LDPE, confirming its resistance to microbial degradation.

Bioaugmentation with *P. guariconensis* significantly enhanced BMF biodegradation. After 168 days, carbon mineralization reached 48% for B360 and 36% for EV in ES, compared to 17% and 6.2% in BS, respectively. Microcosm experiments corroborated these results, showing higher microbial activity and visible film fragmentation in ES. These findings highlight the potential of targeted bioaugmentation to accelerate BMF degradation in agricultural soils.

## Supplementary Information

Below is the link to the electronic supplementary material.

Supplementary file1 (DOCX 9058 KB)

## Data Availability

The authors declare that the data supporting the findings of this study are available within the paper and its Supporting Information files.

## Abbreviations

AMF: Agricultural mulch film(s)
BMF: Biodegradable mulch film(s)
LDPE: Low-density polyethylene
PLA: Poly(lactic acid)
PBAT: Poly(butylene adipate-co-terephthalate)
TPS: Thermoplastic starch
PCL: Poly(caprolactone)
BHM: Bushnell Hass mineral medium
TE: Trace elements
BHM + TE: Bushnell Hass mineral medium with trace elements
TSB: Tryptic soy broth
PCA: Plate count agar
PDA: Potato dextrose agar
PE: Poly (Ethylene)
ES: Enhanced soil
BS: Baseline soil
EV: EcoVio (abbrev. used in figures/text)
B360: Bio360 (abbrev. used in figures/text)
CFU: Colony-forming units
ANOVA: Analysis of variance
Tukey (HSD): Tukey’s honestly significant difference
Fisher’s LSD: Fisher’s least significant difference
PCR: Polymerase chain reaction
16S rRNA: 16S ribosomal RNA
NCBI: National Center for biotechnology information
BLAST: Basic local alignment search tool
YPD: Yeast extract peptone dextrose (medium)
DI water: Deionized water
