# Supplementary material for: Isolation and identification of soil bacteria capable of degrading biodegradable mulch films
Source: Biodegradation. 2025 Nov 20;36(6):123. doi: 10.1007/s10532-025-10223-4 (PMC12634803; doi:10.1007/s10532-025-10223-4)
Supplement: Supplementary file 1 — Supplementary file1 (DOCX 9058 KB) [file 10532_2025_10223_MOESM1_ESM.docx]

**SUPPLIMENTAL INFORMATION**

**Isolating biodegradable mulch plastic degrading soil Bacteria with using enrichment culture technique**

Harshal J. Kansara^1^, Yvan D. Hernandez-Charpak^1^, André O. Hudson^2^, Jeffrey S. Lodge^2^, Thomas A. Trabold^1^, Carlos A. Diaz^3*^

1. Rochester Institute of Technology, Golisano Institute for Sustainability, Rochester, NY, USA

2. Rochester Institute of Technology, Thomas H. Gosnell School of Life Sciences, Rochester, NY USA

3. Rochester Institute of Technology, Department of Packaging and Graphic Media Science, Rochester, NY USA

*Corresponding author

Table S1 - Laboratory analysis of soil used to isolate enrichment cultures performed at Dairy One (Ithaca, New York, USA) [1].


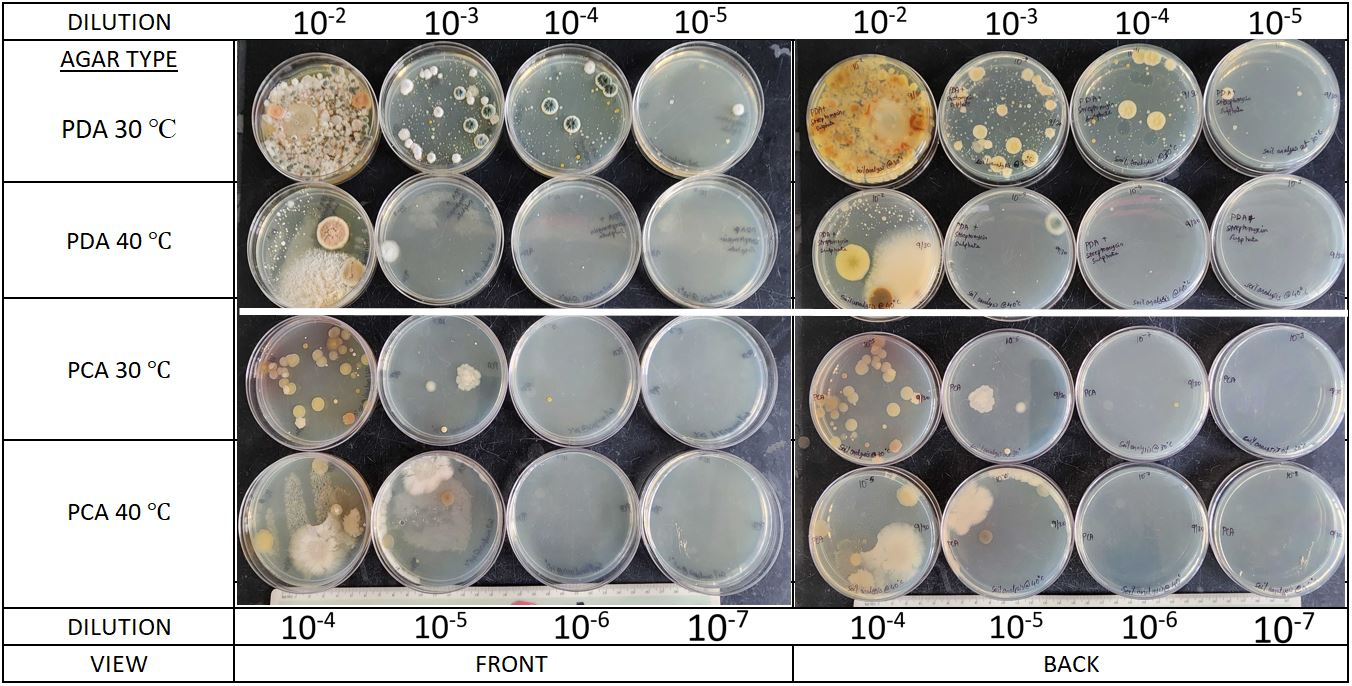


**Figure S1 -** Plate Count Agar (PCA) and Potato dextrose agar (PDA) with streptomycin sulfate (1µg/ml) were used to isolate bacterial and fungal cultures respectively. The incubation was performed at 30℃ and 40℃ for 48 hrs. Significant microbial abundance and diversity can be seen thus ensuring a good soil quality, ideal for enrichment experiments [1].


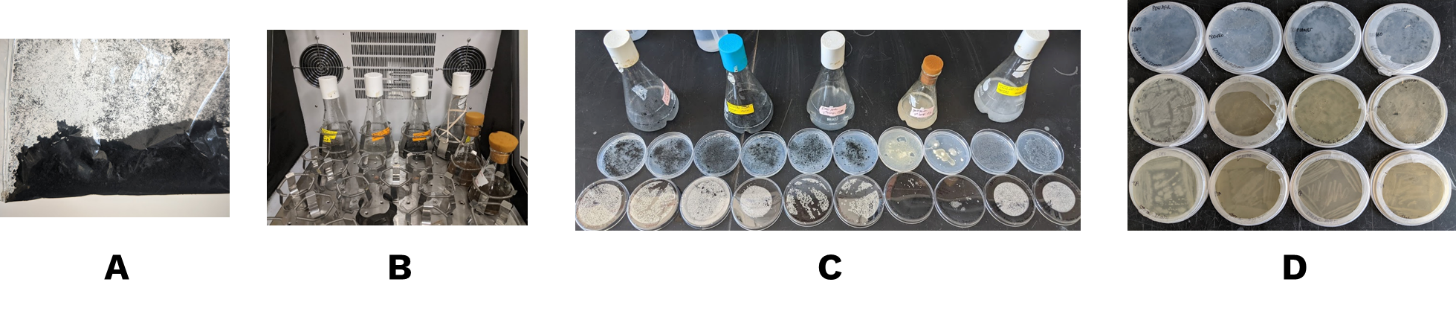


Figure S2 – The enrichment culture method utilized is shown in brief. Cryo-milled Bio360 mulch power is shown in Fig. S2A. Culture enrichment is performed first for 15 days (cycle 1), 20 days (cycle 2) and finally for another 20 days (cycle 3) using shake flasks (fig S2B). Specialized agar plates were created using plastic powders uniformly coated over the surface, inoculated with enriched products and incubated for 7 days (Fig. S2C). The individual cultures were then isolated from the specialized BHM agars using PCA plates (Fig. S2D).


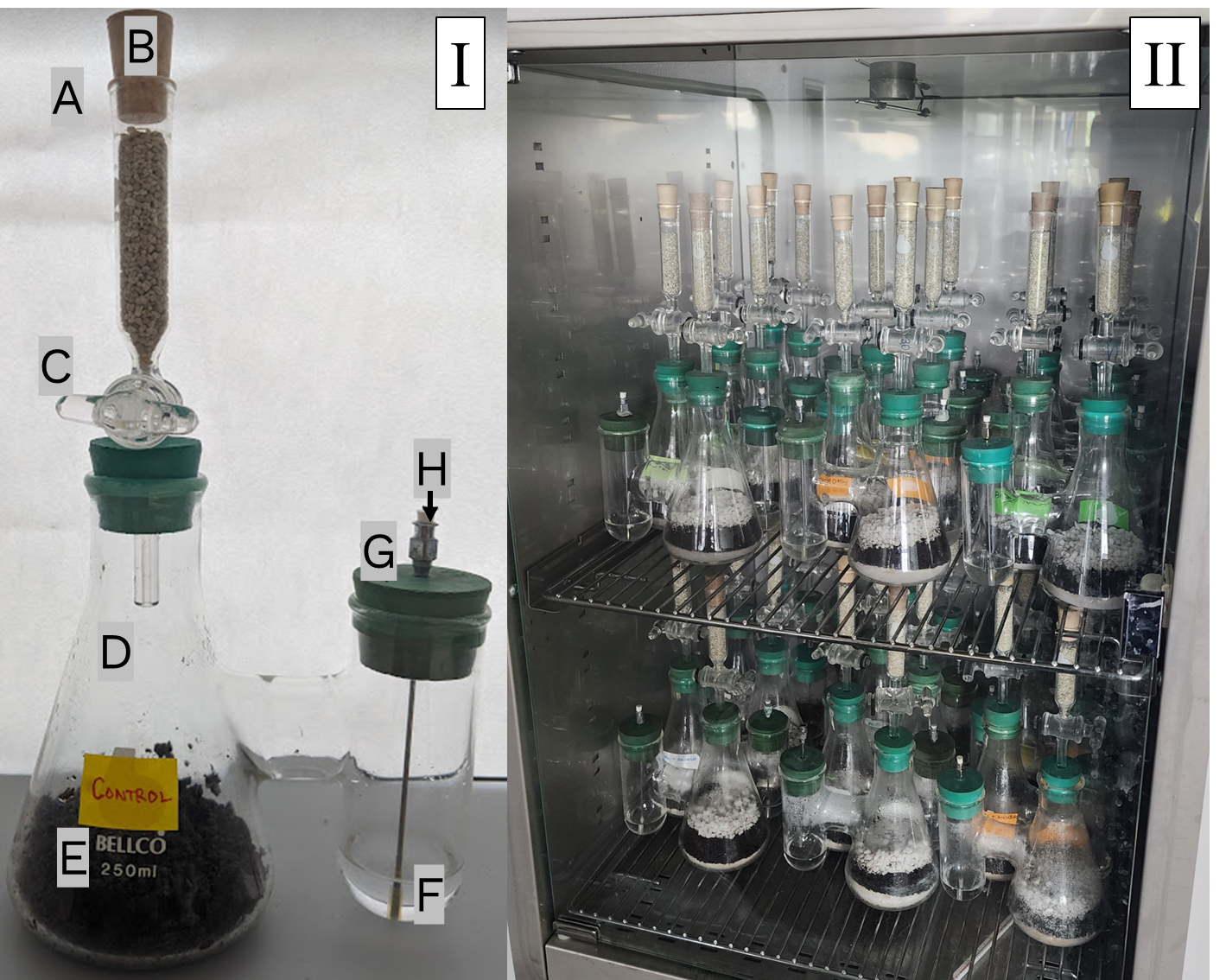


**Figure S3 –** Biometer flask setup is shown in fig S3(I) and final experimental flasks incubated at 30°C are shown in fig. S2(II).

**Setup Procedure:**

Fig. S3-I shows the setup of a typical biometer flask. Ascarite II is packed into tower as shown in (A) and sealed with cork (B). A glass filter stopper (C) is inserted and sealed with an O-ring. Then, 10 g of fine perlite, 10 g of coarse perlite, and 50 g of soil are weighed. The process begins by adding 10 g of fine perlite to the bottom of the flask, followed by transferring 25 g of soil into flask (D). 5 cm^2^"sample is placed on top of the soil. The remaining 25 g of soil is added on top of the sample, followed by 10 g of coarse perlite. For the control sample, 50 g of soil is used without any film substrate. See Fig. S3-II for the final flask setup.

Next, 10 mL of 0.7 M KOH is added to the flask (F) and sealed with a stopcock and needle (G), which is further protected from the ambient CO_2_ in the air with a small rubber cork (H). The KOH is sampled regularly, which also helps introduce fresh O_2_ into the chamber. OpTech-O2 Platinum sensors (not shown in the figure) were attached to the inner wall of the Erlenmeyer part of the flasks to ensure that the soil does not turn anaerobic in nature due to lack of O_2_.

To sample KOH, cork (B) is first removed, followed by turning glass stopper (C) to the open position. The small rubber stopper H is then opened, and KOH is extracted using a syringe and transferred to a 125 mL Erlenmeyer flask. Fresh 10 mL KOH is replenished in the biometer flask. Finally, rubber stopper H, glass stopper C, and cork B are sealed again. This process is repeated for all flasks, including controls. The experiment is conducted in triplicate.

**Titration procedure:**

CO2 readings were obtained by sampling 10 mL of spent KOH from the biometer flask into a 125 mL Erlenmeyer flask. 1–2 drops of 1% phenolphthalein solution were added, turning the solution a transparent purple. HCl was then slowly titrated using a 50 mL burette until the solution became clear, indicating complete neutralization of KOH. The burette reading was recorded, and the process was repeated for all flasks. These readings were then used to generate carbon mineralization curves, following formulas {1} through {7} from *section 2.7.2* of the manuscript.

**
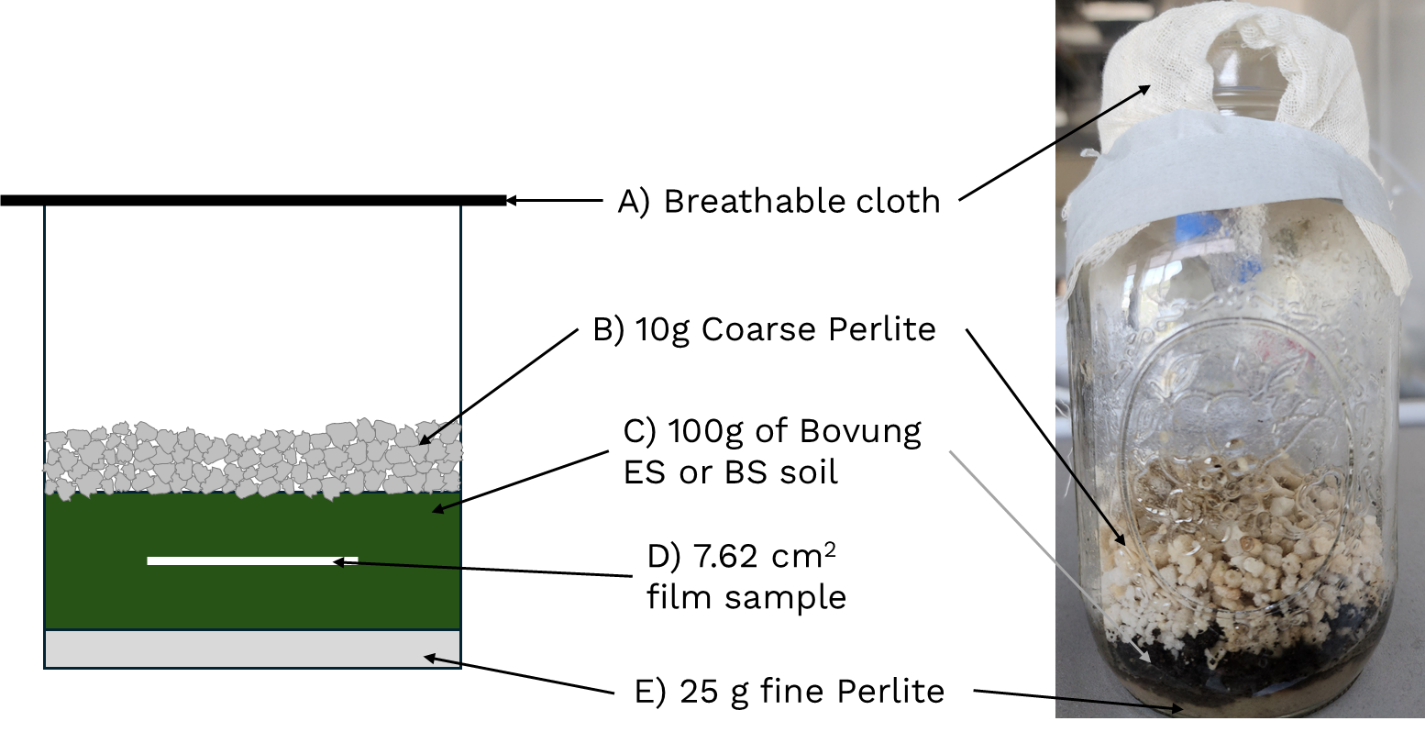
**

**Figure S4 –** An illustration of the jar-based setup used to qualitatively assess plastic degradation was established alongside the biometer flasks. Four single replicates were prepared for each sample in both enhanced soil (ES) and baseline soil (BS).

The experimental setup began by adding 100 g of fine perlite to the base of a clean 1 L jar (Fig. S4-E), followed by 20 mL of deionized water to moisten the perlite. A layer of 50 g of soil was then added, with a 7.62 cm² plastic sample placed on top. This was covered with an additional 50 g of soil (Fig. S4-D, Fig. S4-C), and 10 g of coarse perlite (Fig. S4-B) was added as the final layer, moistened with 10 mL of deionized water. The jar was sealed with breathable cheesecloth, and its total weight recorded. The jars were incubated at 30°C, and any weight loss due to evaporation was regularly monitored and replenished with deionized water to maintain consistent moisture levels. Due to the incubator's size constraints, only single set of jars were prepared for each sampling timepoint, so the final data represent single replicate per time point rather than triplicates. As the plastic samples degraded, films were periodically retrieved, cleaned using a 70% ethanol and 30% deionized water mixture, and wiped gently. They were then rinsed with deionized water, dried overnight at 60°C, and weighed. Additionally, the remaining soil from the microcosm experiments was diluted and subjected to microbial assays on PCA agar to track colony counts over time. However, the limitation in the incubator capacity affected the ability to run more replicates for further validation.


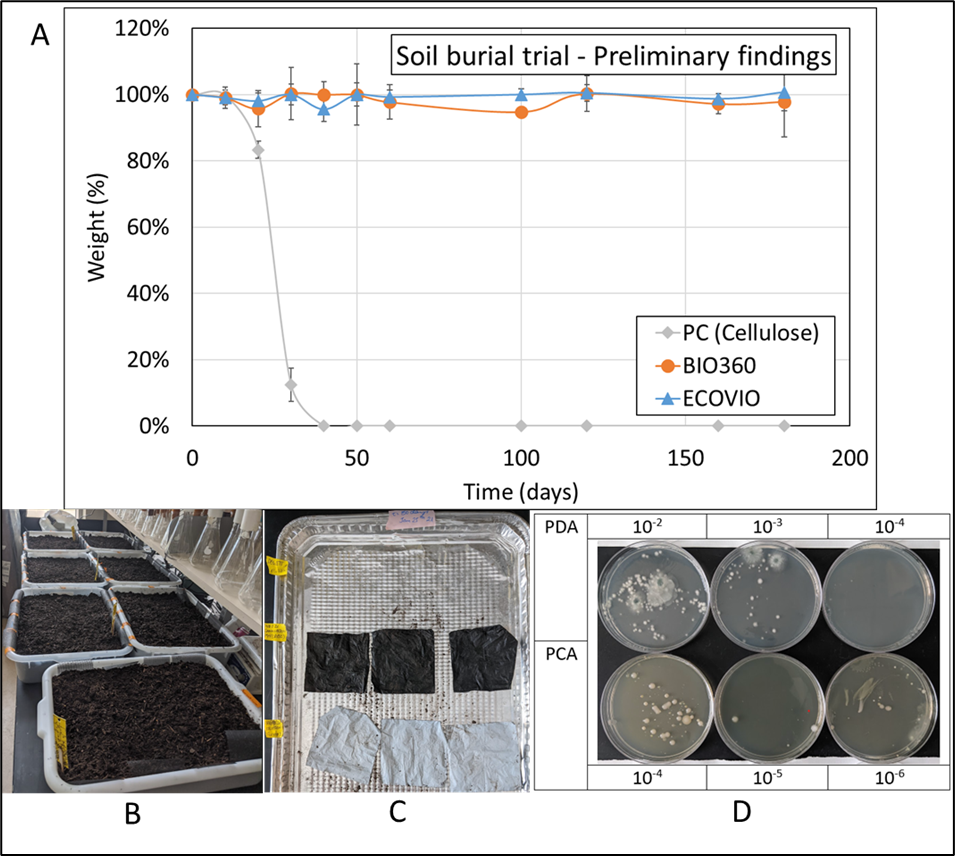


**Figure S5** – Ten identical 5-gallon soil bins, each containing triplicate 3” x 3” Bio306, EcoVio, and cellulose films, were prepared following the schematic in Fig-S1B. The bins were incubated at 21°C ±1°C, and samples were retrieved at regular intervals, with each bin containing samples for a specific time point. The detailed setup of the soil burial trial has been previously described by Bhattacharya et al. [1]. Soil samples were collected from areas surrounding each degrading film throughout the trial, and microbial isolations were performed using the same procedure. Fig-S1D presents an example of microbes isolated from B360 after 150 days of the biodegradation trial on PCA and PDA. Film samples were cleaned using deionized water and then ethanol to remove soil debris. The 150-day incubated and cleaned films are shown in Fig-S1C. Finally, film samples were weighed. PC (cellulose film) fully biodegraded within 40 days (Fig-S1A), while Bio360 and EcoVio samples did not exhibit significant weight loss within 180 days (Fig-S1A). Pristine BMFs are inherently hydrophobic, impeding the enzymatic hydrolysis process and hindering biodegradation, as observed in this preliminary trial.[2].


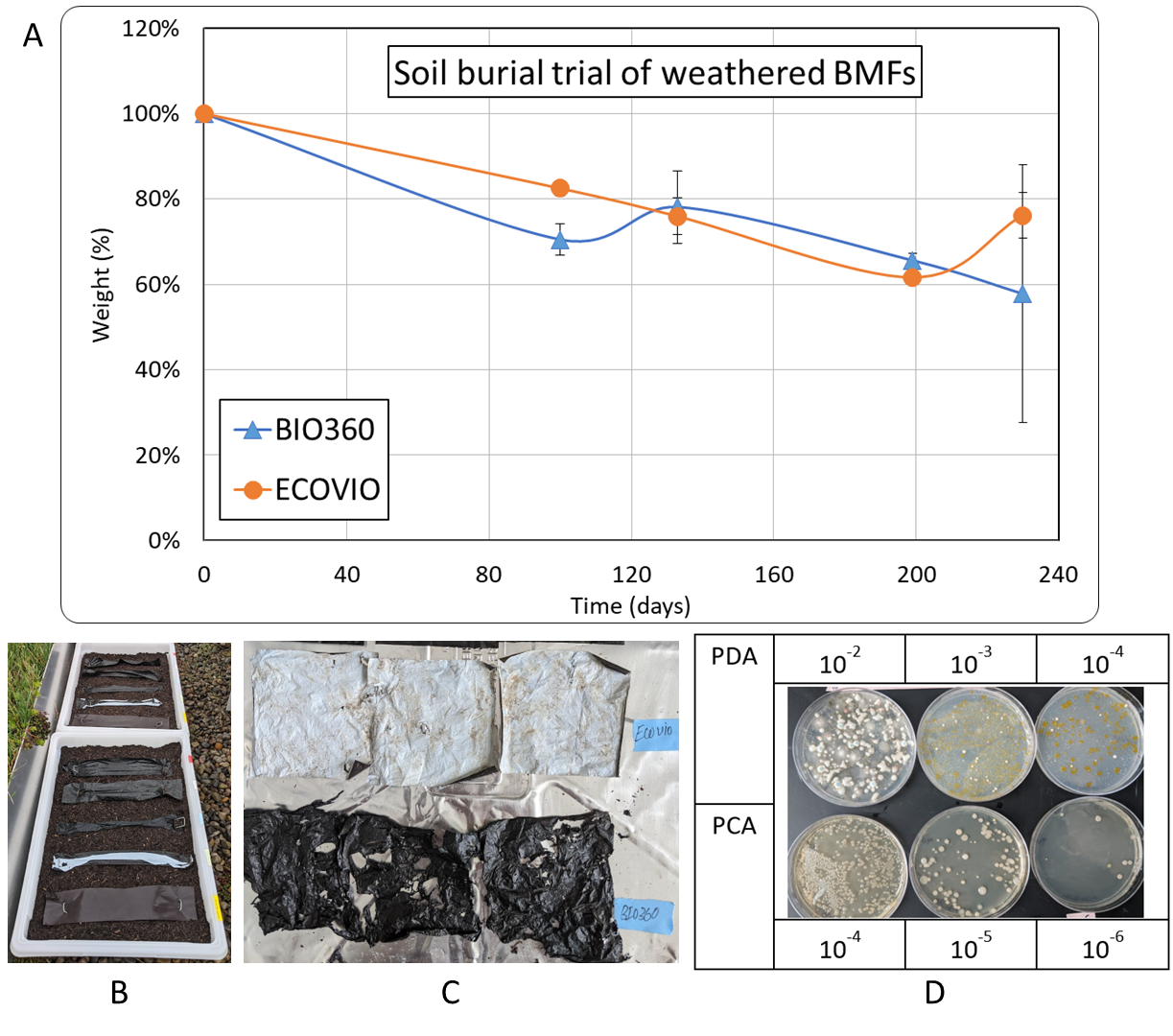


**Figure S6 –** Environmental weathering of BMF samples was conducted in the summer months (June - Aug) to replicate field conditions, as depicted in Fig-S2B. Soil burial trials were then performed on the weathered BMFs using the same experimental parameters outlined in Fig-S1. Fig-S2A illustrates approximately 43% mass loss for Bio360 films and around 24% mass loss for EcoVio films over 230 days of burial. Environmental weathering factors such as heat, sunlight, and moisture contribute to the deterioration of polymeric chains, rendering them more susceptible to enzymatic hydrolysis [3]. Additionally, microbial analysis presented in Fig-S2D revealed greater diversity and higher microbial colony counts in the soils surrounding B360 film taken after 200 days compared to its pristine film counterpart shown in Fig-S1D. Similar microbial trend was observed for EcoVio film (data not shown). Due to the presence of TPS in its composition, Bio360 undergoes significant fragmentation at an accelerated rate compared to EcoVio. A similar trend was observed in literature where it was reported that biodegradation of TPS+PLA blend shows hastened degradation of the films as compared to a pure PLA film counterpart in composts [4].


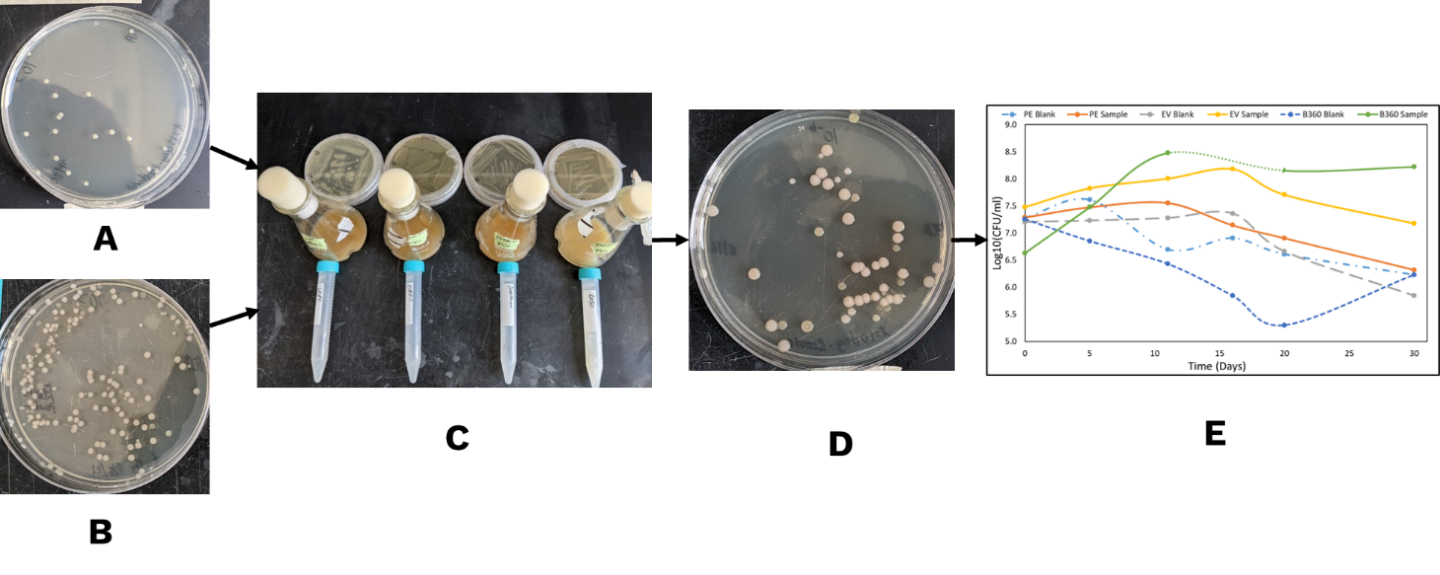


**Figure S7 –** Enrichment of EcoVio mulch led to isolation and identification of *A. denitrificans* (Fig. S4A) and *P. guariconensis* (Fig. S4B). The efficacy that the enriched cultures to grow on the EcoVio as a sole carbon source was tested by first growing individual inoculums in the 25 ml of TBS broth in 125 ml flasks at 30°C for 24 hours (Fig. S4C). 10 ml individual inoculums were then transferred to 15 ml centrifuge tubes (Fig. S4C). Post centrifuge, the cultures were washed with BHM. The cells were then centrifuged and washed twice again. Finally, the cells were re-suspended in 10 ml BHM (Fig. S4C). 500 ml flask containing 125 ml BHM+TE media was taken. 0.5% (w/v) EcoVio plastic powder was added to the flask. 0.5 ml (v/v) washed *A. denitrificans* cells and additionally 0.5% *P. guariconensis* (v/v) cells were inoculated into the 500 ml flask with EcoVio powder. The mixed culture flask was then incubated for 30 days at 30°C. 100 µL of samples were taken every 5 days, diluted and inoculated onto a PCA (Fig. S4D). Figure S4D shows the mixed culture colonies obtained after 25 days of growth. Total colony counts were then taken and plotted as Log(CFU/ml) vs time (Fig. S4E). The same procedure is repeated for isolates from the Bio360 and LDPE mulch. A parallel set of flasks were made with the same quantity of inoculum in the BHM+TE media but no added plastic powder to establish baseline bacterial growth.


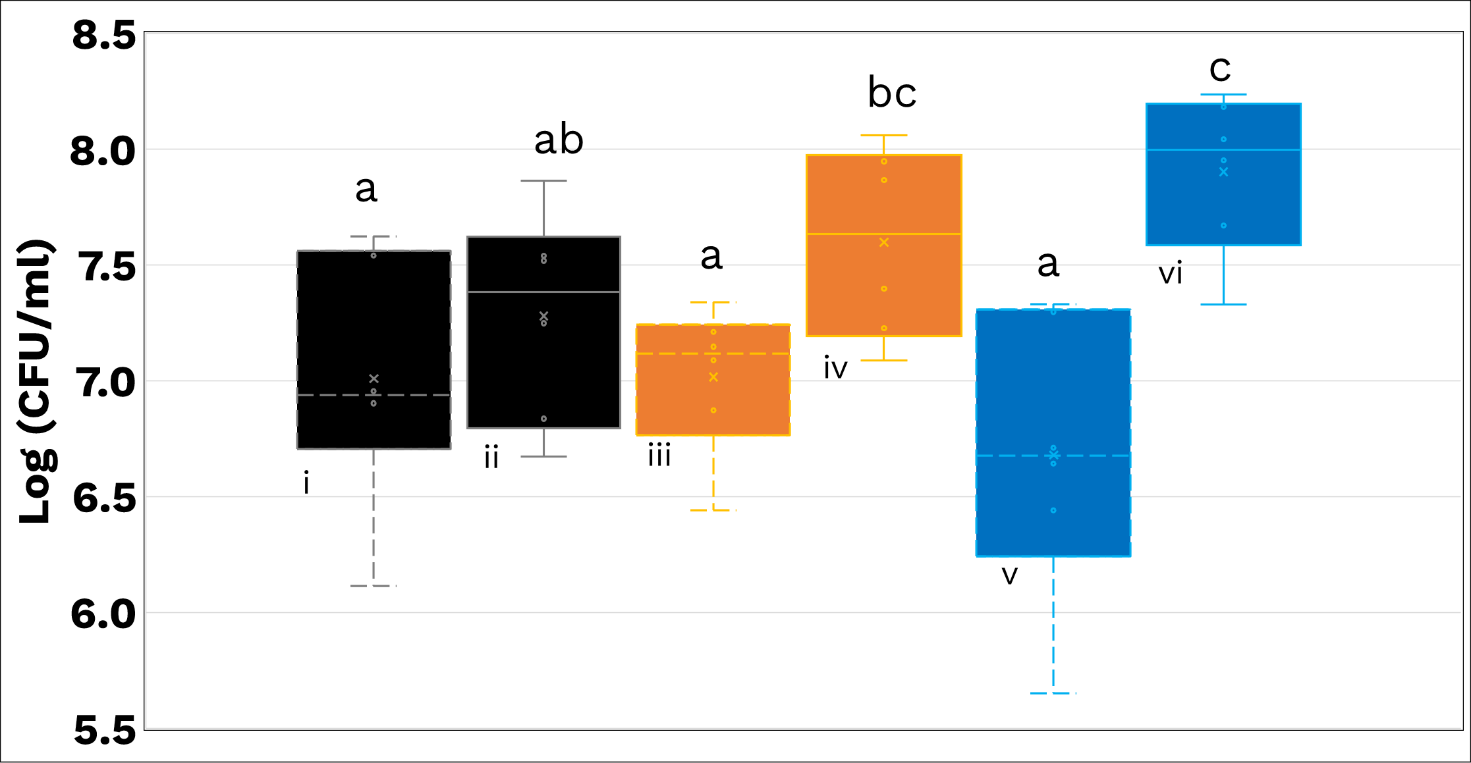


**Figure S8 –** Mixed culture bacterial growth over time was performed using bacteria isolated from BMF enrichment as shown in 2B. Fig 3B shows the mean of the bacterial growth along with the lower and upper hinges of the boxes representing 25th and 75th percentile, whiskers show 1.5 times inter-quartile range and letters displayed represent *Fisher LSD post hoc* results following one-way ANOVA with significance tested at α = 0.05.


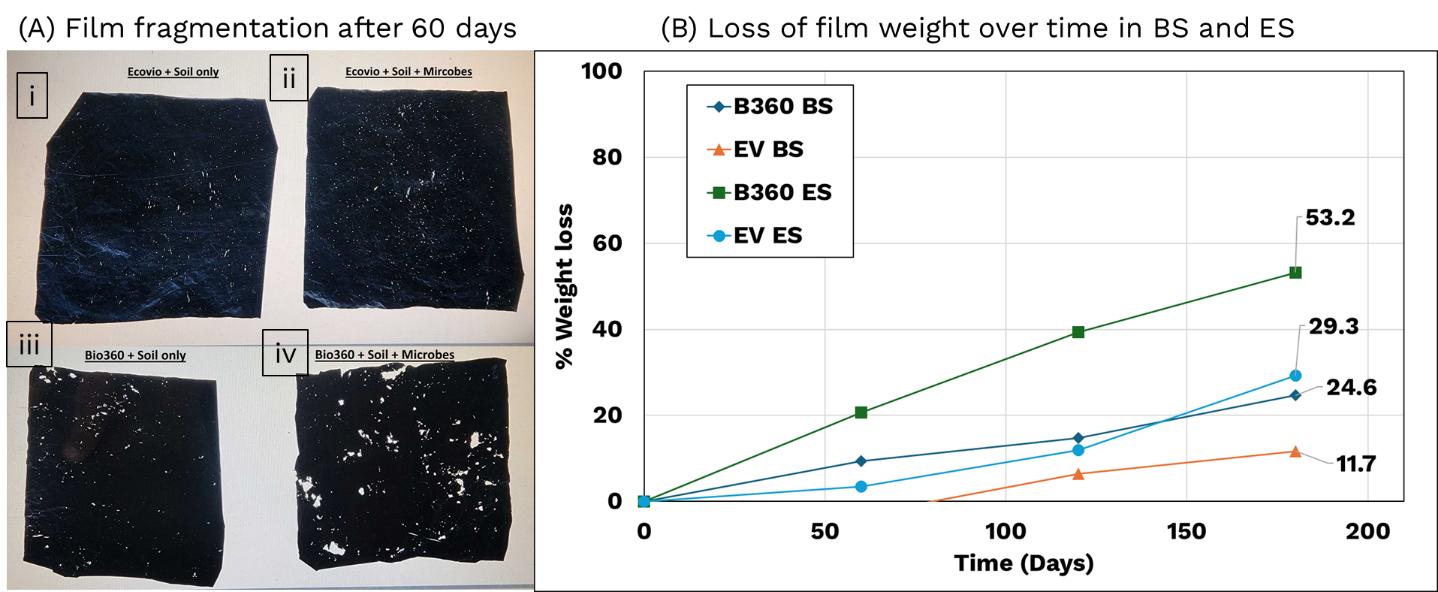
 **Figure S9 –** (A) Images of 60-day film fragmentation from the microcosm experiment under white light, showing (i) EcoVio in Baseline Soil, (ii) EcoVio in Enhanced Soil, (iii) Bio360 in Baseline Soil, and (iv) Bio360 in Enhanced Soil after 60 days of burial. (B) The corresponding weight loss observed over the 180-day microcosm experiment.

The data indicate a clear trend of faster film degradation in Enhanced Soil (ES) compared to Baseline Soil (BS). However, as the data is based on a single replicate, the lack of statistical significance prevents a definitive conclusion.

**Bibliography –**

[1] S. Bhattacharya, H. J. Kansara, J. Lodge, C. A. Diaz, and C. L. Lewis, “Plasma treatment process for accelerating the disintegration of a biodegradable mulch film in soil and compost,” *Front Mater*, vol. 10, p. 1232577, Jul. 2023, doi: 10.3389/FMATS.2023.1232577/BIBTEX.

[2] Y. ; Zhao *et al.*, “Substitution Experiment of Biodegradable Paper Mulching Film and White Plastic Mulching Film in Hexi Oasis Irrigation Area,” *Coatings 2022, Vol. 12, Page 1225*, vol. 12, no. 8, p. 1225, Aug. 2022, doi: 10.3390/COATINGS12081225.

[3] D. G. Hayes *et al.*, “Effect of diverse weathering conditions on the physicochemical properties of biodegradable plastic mulches,” *Polym Test*, vol. 62, pp. 454–467, Sep. 2017, doi: 10.1016/J.POLYMERTESTING.2017.07.027.

[4] P. C. Mayekar, W. Limsukon, A. Bher, and R. Auras, “Breaking It Down: How Thermoplastic Starch Enhances Poly(lactic acid) Biodegradation in Compost─A Comparative Analysis of Reactive Blends,” *ACS Sustain Chem Eng*, vol. 11, no. 26, pp. 9729–9737, Jul. 2023, doi: 10.1021/ACSSUSCHEMENG.3C01676/ASSET/IMAGES/LARGE/SC3C01676_0005.JPEG.
